# Infantile fibrosarcoma of the perineum with dorsal metastasis in a neonate: a case report original

**DOI:** 10.1186/s12887-023-04129-4

**Published:** 2023-06-29

**Authors:** Juan Geng, Dan Chen, Limin Wang, Xiangjiao Liu, Wenjing Chen, Hongyi Gao, Shangjie Xiao

**Affiliations:** 1 Department of Ultrasound, Guangdong Women and Children Hospital, Guangzhou, China; 2 Department of Pathology, Guangdong Women and Children Hospital, Guangzhou, China; 3 Department of Neonatal Surgery, Guangdong Women and Children Hospital, Guangzhou, China

**Keywords:** Infantile fibrosarcoma, Soft tissue tumor, Ultrasonography, Infant

## Abstract

**Background:**

Infantile fibrosarcoma is a rare pediatric soft tissue tumor and usually appears in children before one year of age. Distal extremities constitute the most frequently affected locations, and other tissues such as the trunk, head and neck, gut, sacrococcygeal region, and viscera are uncommon sites.

**Case presentation:**

We describe a rare case of infantile fibrosarcoma arising from the perineum. First, a cystic mass was detected using prenatal ultrasonography, and then an echo was changed in serial ultrasound examinations. A solid cystic lesion was found at term; a hypoechoic lesion occurred in the back. The tumor became so large that massive bleeding occurred, which then underwent surgical resection. Pathological examination confirmed infantile fibrosarcoma.

**Conclusion:**

Our report demonstrates not all ultrasonographic findings in cases of infantile fibrosarcoma exhibit a solid mass during the initial examination — an early-stage lesion may reveal a cystic echo. Infantile fibrosarcoma has a good prognosis and surgery constitute the main treatment, with adjuvant chemotherapy being received if necessary.

## Background

Infantile fibrosarcoma is a rare malignant soft tissue tumor composed of mesenchymal fibroblasts, histologically is characterized by uniform spindle-shaped cells packed in a herringbone pattern. It usually occurs in superficial or deep tissues of distal extremities and then extends to other surrounding soft tissues [1], and other areas such as the head and neck, trunk, gut, sacrococcygeal region, and viscera are uncommon sites [2–5]. As a locally invasive tumor, it has a good prognosis, and the rate of metastasis is less than 10% [6, 7]. Herein, we report a rare case of a neonatal perineal tumor with dorsal metastasis in which pathological findings confirmed infantile fibrosarcoma.

## Case presentation

**Fig. 1:**
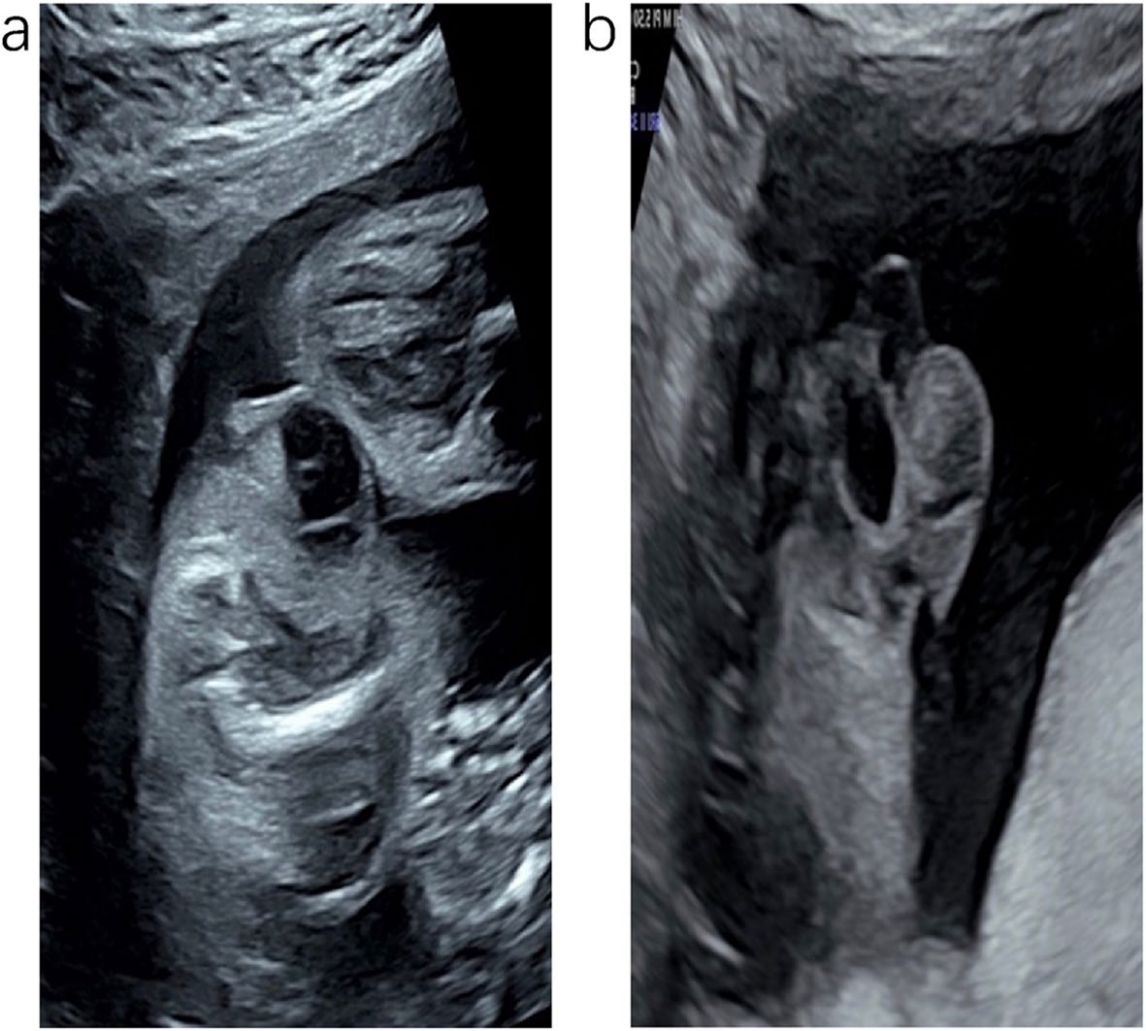
Prenatal ultrasound (**a**) Two-dimensional ultrasound showing a cystic mass in the perineum at 24 gestational weeks. **b** A solid component began to appear in the cystic mass at 27 gestational weeks

**Fig. 2:**
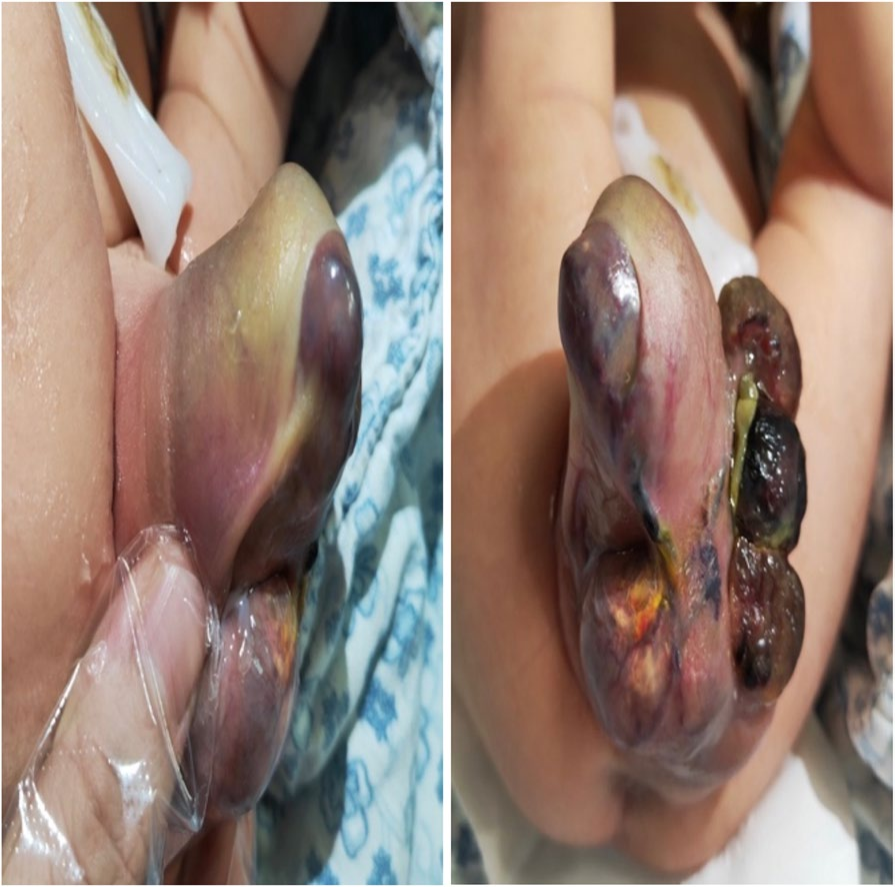
There is a huge, irregular mass in the perineum. The external genitalia is difficult to recognize

**Fig. 3:**
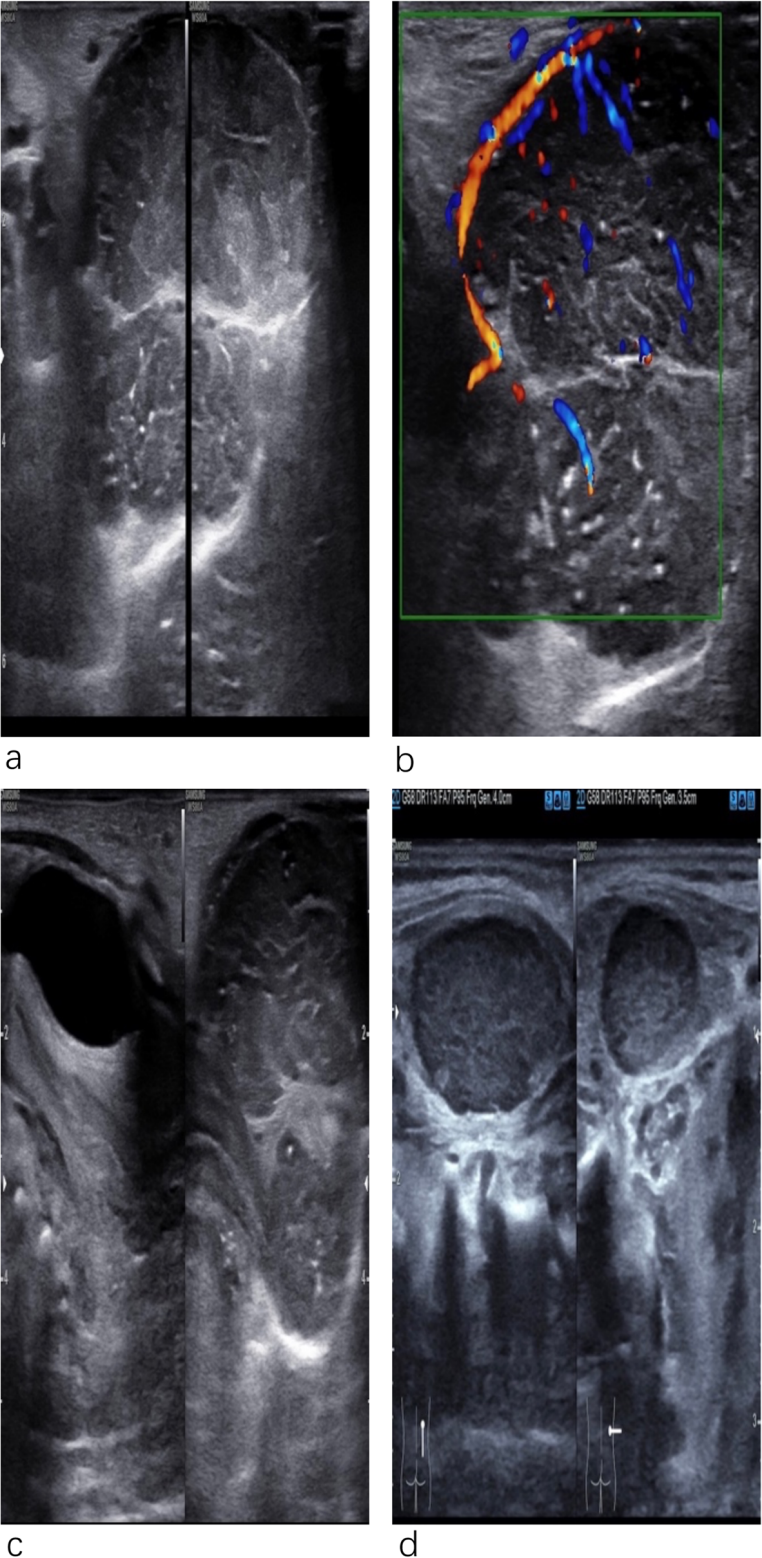
Ultrasound findings of tumor (**a**) Two-dimensional ultrasound showing an irregular hypoechoic solid mass in the perineum. **b** Color Doppler ultrasound showing thick vessels along the edge. **c** Two-dimensional ultrasound showing uterus and the proximal vagina. **d** Two-dimensional ultrasound showing a hypoechoic solid mass in the back

A newborn female infant was referred to our hospital due to a perineal tumor with dorsal metastasis on day 1. At 24 gestational weeks, prenatal ultrasonography depicted a cystic mass in the perineum that measured 2 × 1 cm in size (Fig. 1a). At 27 gestational weeks, the tumor showed rapid development in size (4 × 3 cm), and a solid component began to appear in the cystic mass (Fig. 1b). At 37 gestational weeks, the mass rapidly grew to a size of 6 × 4 cm. A solid cystic lesion was found, and a hypoechoic lesion occurred in the back that measured 3 × 1 cm in size. The pregnancy went very smoothly. No signs of fetal heart failure and fetal edema were visible. At 39 gestational weeks, the baby was born by cesarean section (weight 3000 g). The infant had CT scans of the chest, abdomen, pelvis and brain on postnatal day 4, no abnormal findings were observed except for metastasis in the back. Physical examination: The baby had a huge, irregular mass in the perineum (6 × 5 cm); thus, there was a failure to recognize the external genitalia (Fig. 2). The urethral orifice was visible. A subcutaneous mass in the right back measured 3 × 2 cm in size. When squeezed, it moved easily but continued to maintain its shape without abnormal findings in the overlying skin. Ultrasonography: Ultrasonographic images displayed an irregular hypoechoic solid mass with a clear boundary in the perineum, measuring 7 × 5 cm in size, the internal echo of which was nonuniform with many septations (Fig. 3a). Thick vessels were observed along the edge, which gave off branches through which to supply the inner part of the mass (Fig. 3b) — peak flow speed: 10.37 cm/s, RI 0.61. The uterus was found, and the proximal vagina was evident, except for the distal region (Fig. 3c). In an ultrasound examination, the lesion in the back, measuring 3 × 2 cm in size, was hypoechoic, had morphology with a regular pattern, was well-circumscribed, and had a homogeneous internal echo (Fig. 3d). It was noted that the lesion had blood vessels at their edge — peak flow speed: 4 cm/s, RI 0.67. MRI examination: The lesion was found to be an irregular lobulated heterogeneous signal intensity mass that measured 6 × 5 cm in size. It was isointense to slightly low signals on T1W1 and slightly high signals on T2W1, with hypervascular features. In addition to the unclear boundary with the pelvic floor, the urethra, and the distal vagina, it also invaded the external urethral orifice, the left lateral vaginal wall, the left-sided ischiorectal fossa, and the left gluteals. The infant was diagnosed as having rhabdomyosarcoma via all imaging examinations. The tumor developed ulceration and bleeding with suspected intratumoral bleeding on postnatal day 6, and tumor resection was performed on postnatal day 7 because of the massive bleeding, which was located in the psoas of the right back, as observed in operation, with a capsule measuring 2 × 2 cm in size. The tumor in the perineum was solid, with a capsule measuring 8 × 5 cm in size, the roof of which was connected to the periosteum of the pubis bone. Although the tumor was huge, it was well capsuled to be peeled off completely without losing much blood and any complication during the surgery. Following surgery, the vagina and urethra suffered no injury. Histological findings: The histopathological examination revealed vascular spaces composed of loose spindle-shaped cells mixed with dilated capillaries. The spindle-shaped cells were arranged closely, and focal necrosis was observed. Immunohistochemical analysis: vimentin ( +), CD31 (vasculature +), CD 34 ( +), CD99 ( +), Desmin (-), S-100 (-), SMA (-), CK (-), EMA (-), confirming the diagnosis of infantile fibrosarcoma with dorsal metastasis. ETV6-NTRK3 fusion demonstrated a negative result. The infant did not undergo any adjuvant therapy. She has been stable for six months postoperatively without any evidence of recurrence, but continued observation is recommended.

## Discussion and conclusion

Infantile fibrosarcoma, a rare mesenchymal soft tissue tumor, is one of the non-rhabdomyosarcoma sarcomas, which usually appears in children before one year of age and affects about five neonates per 1 million infants [8]. According to the World Health Organization’s classification of tumors in 2020 [9], the tumor is classified as intermediate (rarely metastasizing). Infantile fibrosarcoma is typically defined as a tumor occurring before two years of age [1]. It has an excellent prognosis, a low risk of metastasis, and a five-year overall survival rate of 80% ~ 90% [7, 10]. This tumor most often occurs in distal extremities in 60% of cases [1, 7, 11], and other sites such as the trunk, head and neck, gut, sacrococcygeal region, and viscera are rare [2–5]. Thus far, there have been no reports on infantile fibrosarcoma of the perineum with dorsal metastasis.

They usually appear on ultrasounds as a well-circumscribed, hypoechoic soft tissue mass with rich blood flow signals [12, 13]. In our study, the echogenicity gradually changed over time. First, a cystic mass was detected, and then echo changed in serial ultrasound examinations. A solid cystic lesion was found at term, which later progressed toward a mixed echogenicity and finally exhibited a solid mass after birth. Despite the initial character during the first ultrasound examination showing a cystic component, multiple examinations described an altered echogenicity, alerting us to the possibility of it being malignant. Thus, not all ultrasonographic findings in cases of infantile fibrosarcoma exhibit a solid mass during the initial examination — an early-stage lesion may reveal a cystic echo. Rhabdomyosarcoma, the most common pediatric soft tissue tumor, frequently occurs in the head, neck, and genitourinary systems. At diagnosis, 10% of patients present with metastasis [10]. The imaging findings amongst infantile fibrosarcoma patients are not specific [14, 15]. Urogenital rhabdomyosarcoma also appears as a hypoechoic mass on an ultrasound [16, 17]; therefore, our case was misdiagnosed as rhabdomyosarcoma.

In the present case, infantile fibrosarcoma is huge and difficult to differentiate from rhabdomyosarcoma; nevertheless, ultrasonography could provide an efficient method for preoperative clinical evaluation. Perineal tumors near the sacrococcygeal region are often misdiagnosed as sacrococcygeal teratoma, one of the most common fetal tumors which arises from pluripotent embryonic stem cells. Sacrococcygeal teratoma typically presents with sacral mass, and on ultrasonography it appears as cystic, solid (most frequently), mixed. The majority grow slowly, but some with abundant blood supply can rapidly grow, which brings on fetal edema, polyhydramnios and fetal heart failure [18, 19]. Sacrococcygeal teratoma could lead to intraspinal nerve impairment, but the structures inside of the spine, as observed via the ultrasound, were normal in this case, and the histopathological examination revealed closely packed spindle-shaped cells. The tumor was so large that the external genitalia was difficult to identify. With the help of ultrasound, the uterus and ovary were easily observed, which classified as biologically female. The ultrasonography clearly showed the bladder and vagina; meanwhile, the lesion was distinctly demarcated. Given these findings, reproductive system tumors were ruled out. Huge perineal tumors may cause associated genital, anorectal and sacral abnormalities such as anal imperforation and cloacal malformations [20, 21]. Clear anal canal structures were demonstrated on the ultrasound. The distal vagina and distal urethra were hardly observed because of the huge size of the tumor. Those findings alerted clinicians to meticulous dissection during resection. Fortunately, the urethra and vagina were intact without damage. In fact, differential diagnosis of infantile fibrosarcoma from other soft tissue tumors such as rhabdomyosarcoma is very difficult, rhabdomyosarcoma is the most common pediatric soft tissue sarcomas which derives from undifferentiated mesenchyme. Regarding the degree of differentiation, it is similar to skeletal muscle, hence immunohistochemistry is positive for desmin [16]. This tumor most often occurs in head and neck, genitourinary tract and extremities, about 10%-20% of children have metastases at the time of diagnosis [10]. The appearance of rhabdomyosarcoma on MRI is not specific, usually demonstrates isointense T1 which resembles the image of infantile fibrosarcoma [16, 22], thus, the final diagnosis is based on pathological examination.

Pathological examination is utilized to diagnose patients with infantile fibrosarcoma [1]. Using microscopy, it was observed that the spindle-shaped cells were arranged closely, and focal necrosis was observed in this case. As reported, ETV6-NTRK3 fusions, developed from t (12;15) (p13; q25) [23], have been observed in about 85% of infantile fibrosarcoma cases. However, if the breakpoint is in an unconventional position or NTRK-variant fusions occur, a false negative outcome is possible [24].  Although our genetic testing for ETV6-NTRK3 fusions was negative, the finding that specific spindle-shaped cells were found during pathological examination did not change our diagnosis.

Infantile fibrosarcoma has a good prognosis, and the rate of metastasis is less than 10% [6, 7]. Daniel Orbach et al. [7] retrospectively analyzed 56 cases of infantile fibrosarcoma from 1979–2005 and reported that one patient with a forearm tumor showed lymph node metastasis, while one patient with an abdominal tumor presented with pulmonary metastasis.

Daisuke Nonaka et al. [25] described how a fetus at 26 weeks of age was diagnosed with infantile fibrosarcoma based on an in-utero needle core biopsy. Liver metastasis and left adrenal gland metastasis were found during the autopsy three weeks later. There have been some reports of metastasis to the central nervous system and bones [26, 27]. What is unique about this case is that the primary tumor was in the perineum, and metastasis was found through prenatal ultrasound. Surgical resection currently is the mainstay of treatment, and when the tumor is large, adjuvant chemotherapy might be required. Infantile fibrosarcoma has a favorable prognosis, and the five-year overall survival rate is as high as 80% ~ 90% [28].

Infantile fibrosarcoma is a rare soft tissue tumor but is locally invasive, having an excellent prognosis and a low transfer rate. Previous reports have demonstrated that the tumor usually appears on ultrasounds as a hypoechoic soft tissue mass. In our study, a cystic mass was detected at first, which later became a solid cystic mass and finally exhibited a solid mass after birth. Although it is rather difficult to differentiate from rhabdomyosarcoma based on imaging studies, ultrasonography plays an important role in making an earlier diagnosis, monitoring the disease process, detecting metastasis, and guiding clinical decision-making.

## Data Availability

All data used during this study are available from the corresponding author on reasonable request.
